# The complete chloroplast genome sequence of *Aconitum coreanum* and *Aconitum carmichaelii* and comparative analysis with other *Aconitum* species

**DOI:** 10.1371/journal.pone.0184257

**Published:** 2017-09-01

**Authors:** Inkyu Park, Wook-jin Kim, Sungyu Yang, Sang-Min Yeo, Hulin Li, Byeong Cheol Moon

**Affiliations:** 1 K-herb Research Center, Korea Institute of Oriental Medicine, Daejeon, Republic of Korea; 2 Department of Agronomy, Yanbian University Agriculture College, Yanji, China; National Cancer Institute, UNITED STATES

## Abstract

*Aconitum* species (belonging to the Ranunculaceae) are well known herbaceous medicinal ingredients and have great economic value in Asian countries. However, there are still limited genomic resources available for *Aconitum* species. In this study, we sequenced the chloroplast (cp) genomes of two *Aconitum* species, *A*. *coreanum* and *A*. *carmichaelii*, using the MiSeq platform. The two *Aconitum* chloroplast genomes were 155,880 and 157,040 bp in length, respectively, and exhibited LSC and SSC regions separated by a pair of inverted repeat regions. Both cp genomes had 38% GC content and contained 131 unique functional genes including 86 protein-coding genes, eight ribosomal RNA genes, and 37 transfer RNA genes. The gene order, content, and orientation of the two *Aconitum* cp genomes exhibited the general structure of angiosperms, and were similar to those of other *Aconitum* species. Comparison of the cp genome structure and gene order with that of other *Aconitum* species revealed general contraction and expansion of the inverted repeat regions and single copy boundary regions. Divergent regions were also identified. In phylogenetic analysis, *Aconitum* species positon among the Ranunculaceae was determined with other family cp genomes in the Ranunculales. We obtained a barcoding target sequence in a divergent region, *ndhC*–*trnV*, and successfully developed a SCAR (sequence characterized amplified region) marker for discrimination of *A*. *coreanum*. Our results provide useful genetic information and a specific barcode for discrimination of *Aconitum* species.

## Introduction

The genus *Aconitum* belongs to the Ranunculaceae and consists of approximately 400 species [1]. These grow in temperate regions and are well known herbaceous medicinal ingredients with great economic value in Asian countries. The dried root tubers of *A*. *carmichaelii* and *A*. *coreanum* have been used as different herbal medicines in Korea and China, namely, Aconiti Lateralis Radix (*Bu-ja* in Korean and *Fu-zi* in Chinese) and Aconiti Koreani Tuber (*Baek-bu ja* in Korean and *Guan-bai-fu* in Chinese), respectively. In addition, the root tubers of *A*. *kusnezoffii*, *A*. *ciliare* (a synonym of *A*. *volubile* var. *pubescens*), and *A*. *triphyllum* (a synonym of *A*. *jaluense* var. *triphyllum*) have also been used as Aconiti Kusnezoffii Tuber in both countries (*Cho-oh* in Korean and *Cao-wu* in Chinese) [2]. The root tubers of *A*. *carmichaelii* have pharmacological effects such as cardiotonic action, impact on blood vessels and blood pressure, anti-arrhythmic effects, anti-inflammation, and analgesic action [3], whereas those of *A*. *coreanum* have anti-arrhythmic, analgesic, and anti-inflammatory activities [4]. The *A*. *kusnezoffii* root tuber (Aconiti Kusnezoffii Tuber) has analgesic, cardiotonic, antioxidant, and immunological activities [5, 6]. Thus, root tubers of *Aconitum* species have been used as a variety of herbal medicines. *Aconitum* plants also contain highly toxic components such as diester-diterpene alkaloids including aconitine, mesaconitine, and hypaconitine [3]. Although they are a widely distributed and important source of medicinal herbs, improvements in the accurate identification of *Aconitum* species are required because it is difficult to determine the species based on the morphological features of plants and medicinal materials.

Chloroplasts (cp) play an important role in photosynthesis and carbon fixation as well as in biosynthesis [7]. The cp genome ranges from 120 to 180 kb in size in higher plants and has a conserved quadripartite structure with a large single copy (LSC) region, a small single copy (SSC) region, and two copies of inverted repeats (IRs) [8]. The cp genome has undergone diverse changes such as changes in size, gene intron gains and losses, expansion/contraction of IRs, and structure rearrangements [9]. However, gene content and orientation are considerably conserved between species [10]. The cp genome consists of 110 to 130 genes with up to 80 unique protein-coding genes, four ribosomal RNAs (rRNAs), and approximately 30 transfer RNAs (tRNAs) [11, 12]. The cp genome has been widely used to understand phylogenetic relationships and to identify useful molecular markers, which are used for DNA barcoding, namely super DNA barcoding and to authenticate and identify herbal medicines [13]. The universal plant DNA barcodes ITS, *matk*, and *rbcL* have been used for the evaluation of phylogenetic relationships [14, 15]. In a previous report, the *psbA*–*trnH* intergenic region showed genetic variation in 134 individuals belonging to 19 taxa of *Aconitum* [16], indicating indicating interspecies variation of more than 85% in the *psbA–trnH* intergenic spacer. Nineteen taxa of *Aconitum* were classified into ten subgroups. However, in *Aconitum* species, more genomic information is required for accurate identification. Although *Aconitum* species are important in herbal medicine, there is little genomic and sequence information available.

The sequence characterized amplified region (SCAR) markers are valuable genetic tools for the authentication of medicinal plants and herbal medicines because they are simple, reliable, and reproducible [13, 17]. The SACR marker was developed based on sequence information obtained using DNA fingerprinting with the random amplified polymorphic DNA (RAPD) method, and has been used to identify closely related species by PCR amplification of specific target regions [18, 19]. Species identification based on SCAR markers does not need DNA subcloning or sequencing and is more applicable to large scale analyses using multiple samples than the DNA barcoding method. Furthermore, the SCAR assay method can easily discriminate contaminants in a single PCR amplification reaction. Because the SCAR assay is easy to perform, yields highly reproducible results, and allows discrimination between genera and/or species, it is a simple and rapid tool for authenticating herbal medicines and identifying species [17]. In a previous study, important medicinal herbs in Korea, *Aralia continentalis* (Araliae Continentalis Radix) and *Angelica biserrata* (Angelicae Pubescentis Radix), were clearly distinguished and tested for adulteration using a SCAR marker. Also, commercial herbal medicines were tested and were confirmed to be derived from refined herbal medicines and to be free of adulteration [13]. Therefore, a SCAR marker is a powerful molecular tool for determining the botanical origin of herbs used in herbal medicine and for detecting adulterants [20–22].

In this study, we completed *de novo* assembly of cp genomes from *A*. *coreanum* and *A*. *carmichaelii* and compared them with those from other *Aconitum* species to discover highly divergent regions. We also developed a SCAR marker, based on a sequence from a variable gene region in cp DNA, that can distinguish *A*. *coreanum* from other *Aconitum* species. The results will be useful for the standardization and quality control of the herbal medicine Aconiti Koreani Tuber, and could help provide important and valuable information for the verification of the evolutional traits and genetic diversity of *A*. *coreanum* and species belonging to the genus *Aconitum* L., as well as for the genetic engineering of these species.

## Materials and methods

### Plant material, DNA extraction and Illumina sequencing

Plant materials were collected from native habitats and a private farming field in Korea. A sample collection permit of the Korean Institute of Oriental Medicine was obtained from Korean National Parks Service for native habitats at June, 2011 (permit number, Korean National Parks Service-1112). The leaves of *A*. *carmichaelii* and *A*. *coreanum* were used for next-generation sequencing (NGS) analysis, and 27 *Aconitum* germplasms were used for validation of the SCAR marker. DNA was extracted using a DNeasy Plant Maxi kit (Qiagen, Valencia, CA, USA) according to the manufacturer’s instructions. Total libraries from *A*. *carmichaelii* and *A*. *coreanum* were generated using the MiSeq platform (Illumina, San Diego, CA, USA) by LabGenomics, Korea.

### Chloroplast genome assembly and annotation

Cp genomes were obtained by *de novo* assembly from the low coverage whole genome sequence derived from the Phyzen pipeline (http://phyzen.com). Paired-end reads (Phred scores of 20 or more) were assembled using CLC genome assembler (ver. 4.06 beta, CLC Inc, Aarhus, Denmark) with an autonomously controlled overlap size parameter of 200 to 600 bp. The principal contigs in the cp genome were retrieved from total contigs using Nucmer [23] with the cp genome sequence of *Aconitum barbatum* var. *puberulum* (KC844054) as the reference sequence. The contigs were ordered based on the results of BLASTZ analysis [24]. Filtered contigs were linked into a single draft sequence by joining the overlapping terminal sequences of contigs. Gene annotation was performed using the DOGMA program [25] and manual curation was performed using BLAST searches. To verify the exact gene region, reference genomes were compared. Circular maps of *A*. *carmichaelii* and *A*. *coreanum* were obtained using OGDRAW [26]. Codon usage and base composition were determined using MEGA6 [27].

### Genome comparison and repeat analysis

mVISTA was used to analyze similarities between eight *Aconitum* species [28]: *A*. *carmichaelii* (KY407560), *A*. *coreanum* (KU318669), *A*. *volubile* (KU556690), *A*. *austrokoreense* (KY407559), *A*. *chiisanense* (NC029829), *A*. *pseudolaeve* (KY407562), *A*. *longecassidatum* (KY407561), and *A*. *barbatum* (KC844054). The boundaries between IR and SC regions were also compared with those of eight *Aconitum* species: *A*. *carmichaelii*, *A*. *coreanum*, *A*. *volubile*, *A*. *austrokoreense*, *A*. *chiisanense*, *A*. *pseudolaeve*, *A*. *longecassidatum*, *A*. *barbatum*, *Nicotiana tabacum* (NC001879) and *Thalictrum coreanum* (KM206568). Tandem repeats were detected using Tandem Repeat Finder [29] using a length of over 20 bp as the default parameter and the identity of repeats was set to over 90%. Simple sequence repeats (SSRs) were detected using MISA (http://pgrc.ipk-gatersleben.de/misa/misa) with a minimum number of repeats of 10, 5, 4, 3, 3 and 3 for mono-, di- tri- tetra- and hexa nucleotides, respectively, as the default parameter. IRs were detected using the Inverted Repeats Finder [30] with default parameters. The IRs were 20 bp or more in length and showed 90% similarity.

### Phylogenetic analysis

A molecular phylogenetic tree was constructed using 70 protein-coding genes from 38 species, and 36 complete cp genomes were downloaded from GenBank (S1 Table). To identify the phylogenetic position of *Aconitum* species in the Ranunculaes, 20 cp genomes of 13 *Aconitum* species, seven from other species of Ranunclaceae, and 8 species of five other families were used with *Nicotian tabacum* (NC001879) and *Arabidopsis thaliana* (AP000423) as the outgroup. Seventy protein-coding genes were aligned using MAFFT. (http://mafft.cbrc.jp/alignment/server/). Maximum likelihood analysis was performed using MEGA6 with 1,000 bootstrap replicates [27].

### Development of a SCAR marker for *A*. *coreanum*

Highly divergent regions between eight *Aconitum* species were confirmed based on mVISTA similarities, and primers were designed using Primer-BLAST (NCBI). To amplify divergent regions, about 20 ng genomic DNA was added to 20 ul PCR mixture (Solg^™^ 2× Taq PCR smart mix 1, Solgent, Daejeon, Korea) containing 10 pmol primers (Bioneer, Daejeon, Korea). Amplification was performed on a Pro Flex PCR system (Applied Biosystems, Waltham, MA, USA) according to the following step-cycle program: initial denaturing step at 95°C for 2 min; 35 cycles at 95°C for 40 s, 55°C for 30 s, and 72°C for 90 s; and a final extension at 72°C for 5 min. The PCR products were separated on a 2% agarose gel for 40 min at 150 V. Amplified DNA fragments were extracted from agarose gels using a Gel Extraction Kit (Qiagen, Valencia, CA, USA) and subcloned into the pGEM-T Easy vector (Promega, Madison, WI, USA). Inserted DNA was sequenced using a DNA sequence analyzer (ABI 3730, Applied Biosystems Inc., Foster City, CA, USA). NGS data and Sanger sequencing data were aligned using CLUSTALW by Bioedit [31]. *A*. *coreanum*-specific primers were designed for amplification of the SCAR marker. PCR reactions were performed with *A*. *coreanum*-specific primers. Amplification conditions were as follows: 95°C for 2 min; 35 cycles at 95°C for 30 s, 63°C for 30 s, and 72°C for 30 s; and a final extension at 72°C for 5 min. Amplified fragments were verified using 1.5% agarose gel electrophoresis.

## Results and discussion

### Complete chloroplast genome sequence of two *Aconitum* species

Illumina sequencing of paired-end libraries revealed approximately 2.84 Gb of trimmed reads from *A*. *carmichaelii* and *A*. *coreanum* (S2 Table). From aligned contigs against the reference cp genome, we obtained five and ten contigs covering the whole chloroplast genome sequences of *A*. *carmichaelii* and *A*. *coreanum*, respectively (S1 Fig). Single circular sequence was completed after gap filling and manual editing. The complete circular chloroplast genomes of *A*. *carmichaelii* and *A*. *coreanum* were 155,880 bp and 157,040 bp, respectively (S3 Table). Complete cp genomes were compared with the *A*. *barbatum* reference genome [32] using BLASTZ analysis (S1A Fig). We also confirmed by read mapping that the average read mapping depth was 206.52× in *A*. *carmichaelii* and 268.92× in *A*. *coreanum* (S1B Fig). Furthermore, several divergent regions were confirmed by Sanger sequencing. To perform efficient cp genome sequencing, we used low coverage whole genome sequencing with a reference genome sequence [33]. This approach requires less time and has a lower cost than the previously used method [33, 34].

**Fig 1:**
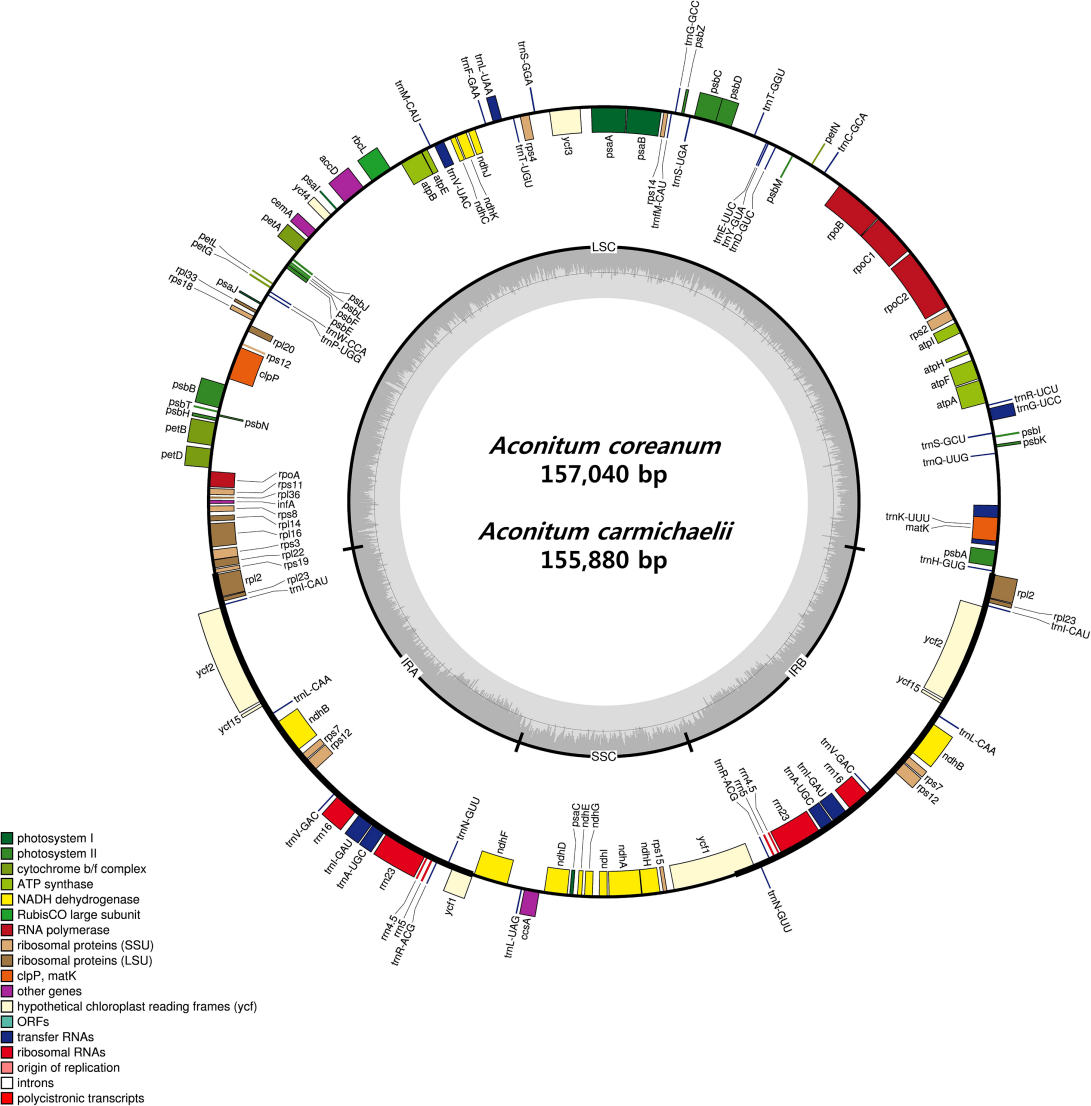
Circular gene map of the two *Aconitum* species. Genes drawn inside the circle are transcribed clockwise, and those outside the circle are transcribed counterclockwise. The darker gray in the inner circle corresponds to GC content.

Both *A*. *carmichaelii* and *A*. *coreanum* chloroplast genomes had the quadripartite structure found in most land plants consisting of a pair of IRs (52,586 and 52,488 bp, respectively) separated by LSC (86,348 and 87,628 bp) and SSC (16,946 and 19,924 bp) regions (Fig 1 and Table 1). The genomes are similar to those of *A*. *barbatum* (156,749 bp) and *A*. *chiisanense* (155,934 bp). The total GC content was 38.13% and 37.99% for *A*. *carmichaelii* and *A*. *coreanum*, respectively, which is similar to published *Aconitum* species [32, 35]. The GC content of the IRs (42.98%, 43.02%) was higher than that of LSC (36.25%, 36.02%) and SSC (32.69%, 32.66%) regions in both *A*. *carmichaelii* and *A*. *coreanum* chloroplast genomes. The IR region showed a higher GC content due to the presence of four rRNA (*rrn4*.*5*, *rrn5*, *rrn16*, and *rrn23*) sequences. Previous studies have reported that high GC content in IR regions is due to the presence of rRNA [36, 37]. The *Aconitum* genomes were AT-rich (61.87% in *A*. *carmichaelii*, 62.01% in *A*. *coreanum*). The chloroplast genomes of *A*. *carmichaelii* and *A*. *coreanum* encoded a total of 112 unique genes, of which 18 were duplicated in IR regions. Among the 112 genes, there were 78 protein-coding genes, four rRNA genes, and 30 tRNA genes (Table 1 and Table 2).

**Table 1:** Size comparison of two *Aconitum* chloroplast genomic regions.

Species	*A*. *camichaelii*	*A*. *coreanum*
Total cp genome size (bp)	155,880	157,040
Large single copy (LSC) region (bp)	86,348	87,628
Inverted repeat (IR) region (bp)	52,586	52,488
Small single copy (SSC) region (bp)	16,946	16,924
GC content (%)	38.13	37.99
LSC (%)	36.25	36.02
IR (%)	42.98	43.02
SSC (%)	32.69	32.66
Total number of genes	131	131
Protein-coding gene	86	86
rRNA	8	8
tRNA	37	37

**Table 2:** Genes present in the two *Aconitum* chloroplast genomes.

Gene products of *Aconitum* species	
Photosystem I	psaA, B, C, I, J
Photosystem II	psbA, B, C, D, E, F, H, I, J, K, L, M, N, T, Z
Cytochrome b6_f	petA, B^1)^, D^1)^, G, L, N
ATP synthase	atpA, B, E, F^1)^, H, I
Rubisco	rbcL
NADH oxidoreductase	ndhA^1)^, B^1)^ ^3)^, C, D, E, F, G, H, I, J, K
Large subunit ribosomal proteins	rpl2^1)^ ^3)^, 14, 16^1)^, 20, 22, 23^3)^, 32, 33, 36
Small subunit ribosomal proteins	rps2, 3, 4, 7^3)^, 8, 11, 12 ^2)^ ^3)^ ^4)^, 14, 15, 18, 19
RNA polymerase	rpoA, B, C1^1)^, C2
Unknown function protein-coding gene	ycf1^3)^, 2^3)^, 3^2)^, 4
Other genes	accD, ccsA, cemA, clpP^2)^, infA, matK
Ribosomal RNAs	rrn16^3)^, rrn23^3)^, rrn4.5^3)^, rrn5^3)^
Transfer RNAs	trnA-UGC^1)^ ^3)^, trnC-GCA, trnD-GUC, trnE-UUC, trnF-GAA, trnG-UCC^1)^, trnG-GCC, trnH-GUG, trnI-CAU^3)^, trnI-GAU^1)^ ^3)^ trnK-UUU^1)^, trnL-UAA^1)^, trnL-UAG, trnL-CAA^3)^, trnM-CAU, trnfM-CAU, trnN-GUU^3)^, trnP-UGG, trnQ-UUG, trnR-ACG^3)^, trnR-UCU, trnS-GCU, trnS-GGA, trnS-UGA, trnT-GGU, trnT-UGU, trnV-UAC^1)^, trnV-GAC^3)^, trnW-CCA, trnY-GUA

1) Gene containing a single intron

2) gene containing two introns

3) two gene copies in IRs

4) Trans-spliced gene.

The two *Aconitum* chloroplast genomes had 17 intron-containing genes, among which 14 (eight protein-coding and six tRNA genes) had a single intron and two (*ycf3*, *clpP*) had two introns (S4 Table). Twelve genes were located in the LSC region (nine protein-coding and three tRNA genes), a protein-coding gene in the SSC region, and four genes in the IR region (two protein-coding and two tRNA genes). The *rps12* gene was trans-spliced because its 5’ end was located in the LSC region and its duplicated 3’ end in the IR region [38]. We also detected the non-canonical start codon ACG in two genes, *rpl2* and *ndhD*. The *Aconitum* cp genomes consisted of 51.05% and 50.59% protein-coding genes (79,590 bp in *A*. *carmichaelii* and 79,461 bp in *A*. *coreanum*), 1.8% and 1.79% tRNA (2,813 bp in both species), and 5.8% and 5.76% rRNA (9,050 bp in both species). The remaining regions were non-coding sequences with intergenic spacers, introns, and pseudogenes. Two pseudogenes were identified: *rps16* had one exon deletion in both *A*. *carmichaelii* and *A*. *coreanum*. The *ycf1* pseudogene was located in the boundary region between IRa and SSC. The codon usage and recognition pattern of the cp genomes are summarized in S5 Table. The 30 unique tRNA genes encode all 20 amino acids essential for protein biosynthesis. A total of 26,530 codons and 26,487 codons were found in *A*. *carmichael*ii and *A*. *coreanum*, respectively (S5 Table). Among these codons, those coding for leucine and isoleucine were the most common in both *Aconitum* cp genomes. In the CDS regions, the percentage AT content at each codon position was 54.6%, 61.4% and 69.6% in *A*. *carmichaelii* and 54.6% 61.4% and 69.7% in *A*. *coreanum*. There was a bias towards a higher AT content at the third position of the CDS, as observed for other *Aconitum* genomes (S6 Table) [39].

### Repeat analysis

Repeat sequences were abundant and diverse in the two genomes, where they play an important role in gene duplication, gene expansion, and DNA rearrangement [40]. Repeat sequences in the *Aconitum* chloroplast genomes were analyzed using MISA and tandem repeat finder. We detected 18 tandem repeats of over 20 bp in length in both *Aconitum* cp genomes (S7 Table). The tandem repeats were 26–54 bp in length. Of these, 44.4% were in intergenic spaces, 38.8% in exons, and 16.6% in introns. Most of the tandem repeats (66.6%) were located in the LSC region. The longest repeat (54 bp) was located in *ycf2* in both *Aconitum* cp genomes. Among the coding regions, the most tandem repeats were present in the *ycf2* gene, which included three tandem repeats. The *ycf1* and *ycf2* genes are associated with a divergent region with many repeat events [41, 42]. We also detected palindromic repeats in both cp genomes. Seven and six palindromic repeats were identified in *A*. *carmichaelii* and *A*. *coreanum*, respectively (Table 3). Among these, six repeats were shared between the two *Aconitum* cp genomes, and were located in the LSC (four repeats) and IR (two repeats) regions. The length of repeats ranged from 21 to 36 bp.

**Table 3:** Palindromic repeats in the two *Aconitum* cp genomes.

Spbcies	Position^a^	Repeat unit length (bp)	Repeat unit sequences	Region^b^
*A*. *carmichaelii*	IGS (*trnE-UUC*, *trnT-GGU*)	23	TTATTTCTATATATTCTAATGAT	LSC
	IGS (*petA*, *psbJ*)	36	GTAAGAATAAGAACTCAATGGAC CTTGCCCCTCGAA	LSC
	IGS (*psbT*, *psbN*)	26	TTGAAGTAAAGTAATGAGCCTCCCAT	LSC
	IGS (*petD*, *rpoA*)	24	ATGTATCTAGGGACTAGTCGCTTC	LSC
	Exon (*ycf2*)	24	AGATCCATTAGATAATGAACTATT	IR
	Exon (*ycf15*)	21	TGGTTGTTCGCCGTTCAAGAA	IR
	Exon (*ycf1*)	25	CTTGATTTAGCGAATCTAAATCAAG	SSC
*A*. *coreanum*	IGS (*trnE-UUC*, *trnT-GGU*)	23	TTATTTCTATATATTCTAATGAT	LSC
	IGS (*petA*, *psbJ*)	36	GTAAGAATAAGAACTCAATGGACCTTG CCCCTCAAA	LSC
	IGS (*psbT*, *psbN*)	28	TTGAAGTAAAGTAATGAGCCTCC-ATAT	LSC
	IGS (*petD*, *rpoA*)	24	ATGTATCTAGGGACTAGTCGCTTC	LSC
	Exon (*ycf2*)	24	AGATCCATTAGATAATGAACTATT	IR
	Exon (*ycf15*)	21	TGGTTGTTCGCCGTTCAAGAA	IR

^a^ IGS, Intergenic sequence

^b^ LSC, Large single copy; IR, Inverted repeat region; SSC, Small single copy.

**Fig 2:**
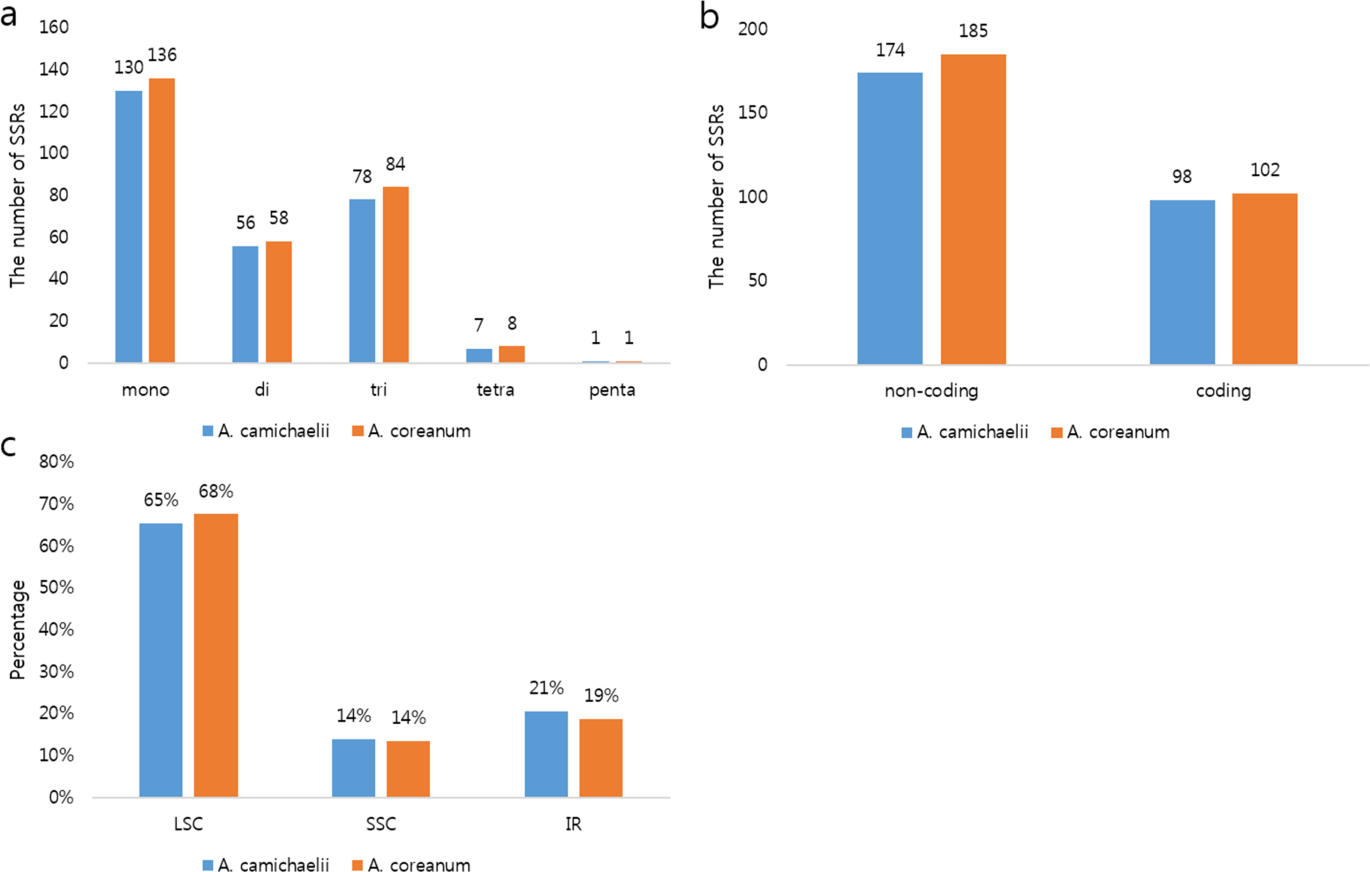
Distribution of SSRs in the two *Aconitum* cp genomes. (A) SSR type distribution in the two cp genomes. (B) The proportion of SSRs in different regions of the *Aconitum* cp genomes. (C) SSR distribution between coding and non-coding regions.

**Fig 3:**
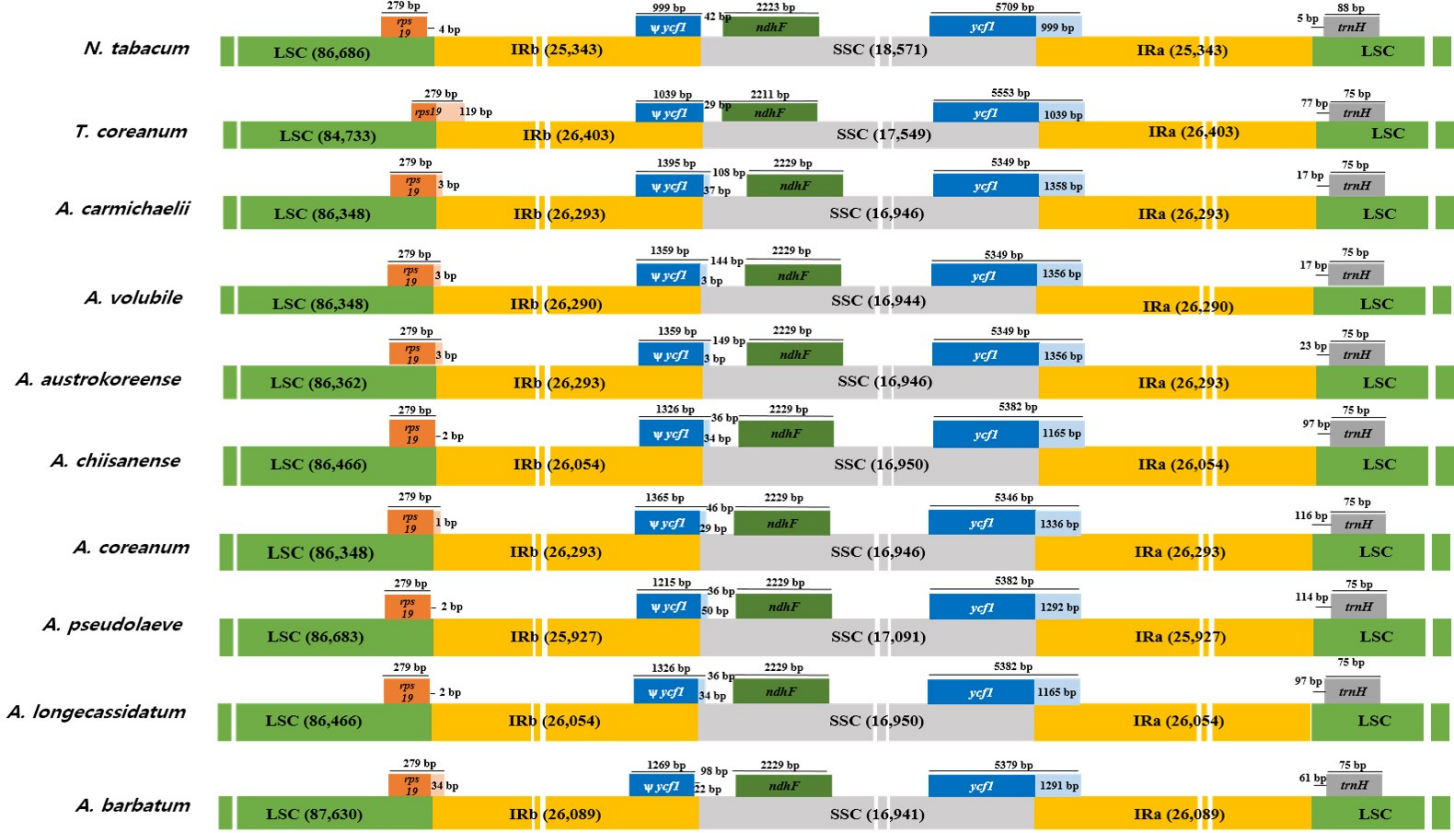
Schematic representations of LSC, SSC, and IR border regions in the eight *Aconitum* species as well as in *N*. *tabacum* and *T*. *coreanum*.

SSRs, or microsatellites, are repeating sequences of 1–6 nucleotides and are widely distributed in genomes [36]. They are easily identified in whole genome sequences and are used as molecular markers due to high interspecies polymorphism, locus-specific co-dominance, and high transferability [43]. We identified 272 and 287 SSRs in the cp genomes of *A*. *carmichaelii* and *A*. *coreanum*, respectively (Fig 2). No hexa-nucleotide repeats were found. Mononucleotide motifs were the most abundant and trinucleotide motifs the second most abundant type of repeat in both *Aconitum* genomes (Fig 2A). A and T repeat units occupied the highest portion due to short polyamine (poly A) or polythymine (poly T) repeats [39]. Furthermore, these SSRs contributed to the AT richness of the *Aconitum* cp genomes. The SSRs were more abundant in non-coding than in coding regions: 64% of all SSRs in both genomes resided in non-coding regions (Fig 2B). Most SSRs were located in the LSC region (65% in *A*. *carmichaelii* and 68% in *A*. *coreanum*). The numbers of SSRs in SSC and IR regions were similar (Fig 3C). These SSRs could be widely used as molecular markers for discrimination of species as well as for genetic diversity and evolution studies.

### Comparison of chloroplast genomes with those of other *Aconitum* species

The two *Aconitum* cp genomes had 98.1% sequence identity and their genome structures, gene contents, and gene orders were similar. The cp genomes of *Aconitum* species are greatly conserved with regard to genome structure and gene orientation and length. Rearrangement such as translocation and inversion was not detected in the two genomes. However, the LSC region of *A*. *coreanum* was 1,280 bp longer than that of *A*. *carmichaelii*, which is similar to that of other plant genomes [32, 35].

Although the IR regions are highly conserved, the expansion and contraction of IR regions are a general feature of chloroplast genomes, where they are mainly responsible for variations in cp genome size and rearrangement [8, 44]. We analyzed the border structures of eight *Aconitum* cp genomes and compared them with those of *T*. *coreanum* [45] and *N*. *tabacum* [46] cp genomes (Fig 3). The lengths of the IR regions of *Aconitum* species were similar and ranged from 25,927 to 26,293 bp, but the expansion and contraction of IR regions differed. The *rps19* genes of three species (*A*. *chiisanense*, *A*. *pseudolaeve*, and *A*. *longecassidatum*) were located exclusively in LSC regions; those of other *Aconitum* species extended into the IR regions. The *ycf1* genes were located at the junctions of IRb/SSC and SSC/IRa border. IRb/SSC extended into *ycf1* genes in all *Aconitum* species except for *A*. *barbatum*. The length of *ѱycf1* ranged from 1,215 to 1,395 bp in *Aconitum* species. The *ycf1* gene was embedded at the IRa/SSC border. The *trnH* genes were all located in LSC regions, 17–114 bp away from the IRa/LSC boundary (Fig 3). The cp genomes are probably preserved in closely related species; species belonging to other families might exhibit considerable variation in their cp genomes, for example, in gene orientation and size, causing expansion and contraction of IR regions.

**Fig 4:**
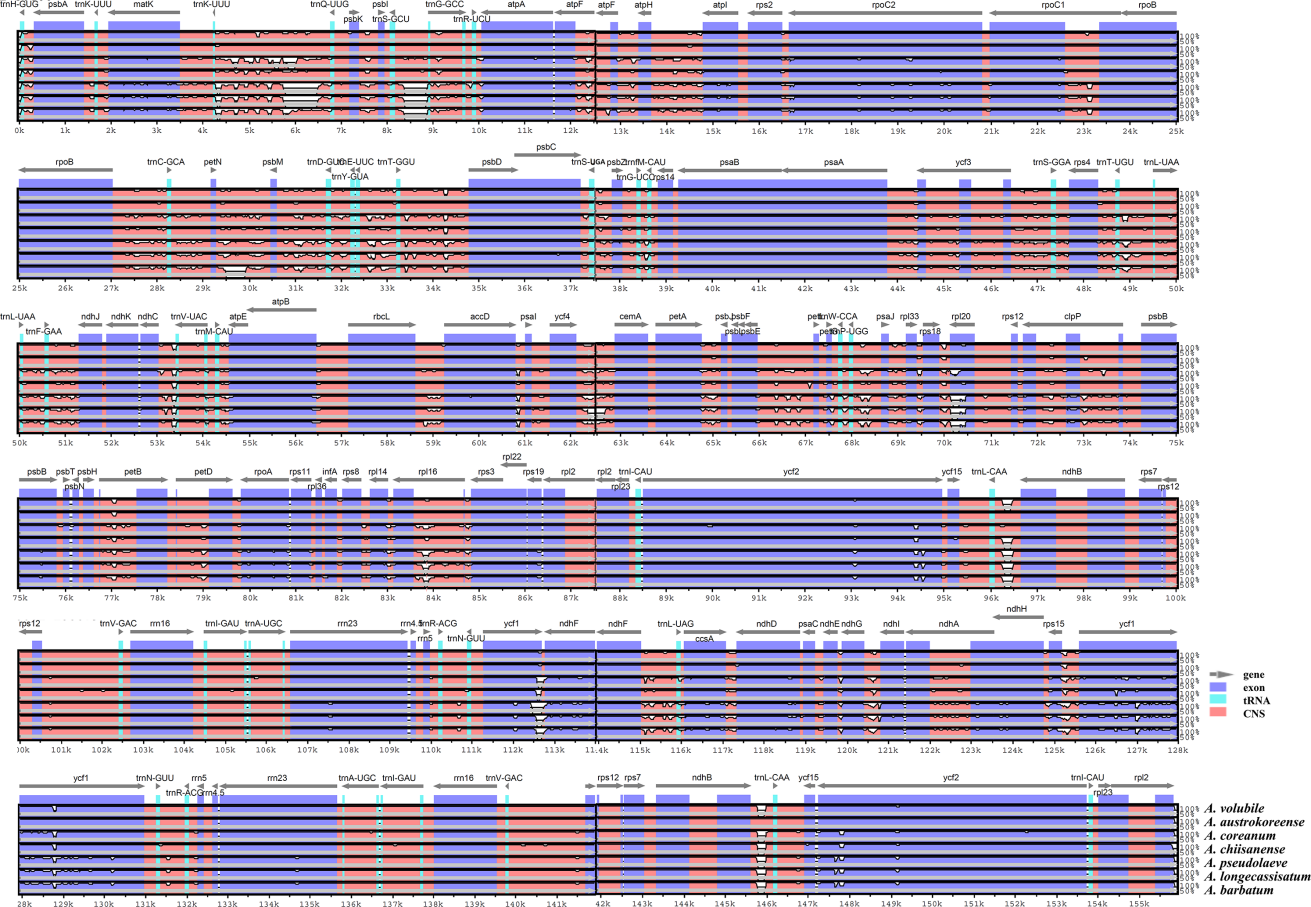
Comparison of eight *Aconitum* chloroplast genomes using mVISTA. Complete cp genomes of eight *Aconitum* species were used for comparison. Genic regions were identified using the DOGMA program, and a comparative map was prepared using mVISTA. Blue block, conserved gene; sky-blue block, tRNA and rRNA; red block, intergenic region. White peaks indicate the regions with sequence variation among *Aconitum* species.

The overall sequences of eight *Aconitum* chloroplast genomes were represented using mVISTA with *A*. *carmichaelii* as reference (Fig 4). The results show that the *Aconitum* chloroplast genomes share highly conserved identity. IR regions were more conserved than other regions due to copy correction by gene conversion following mutation in the IR region [47]. These results are generally consistent with the phylogenetic tree of *Aconitum* species. As expected, the IR regions were more conserved than the LSC and SSC regions. Furthermore, non-coding regions were more divergent than coding regions. In our alignment, the most divergent regions were found in *trnK-UUU*–*trnQ-UUG*, *trnS-GCU*–*trnG-GCC*, *petN*–*psbM*, *ndhC*–*trnV*-*UAC*, *ycf4*–*cemA*, *rpl18*–*rpl20*, and *trnT*–*psbD* regions. These intergenic regions have been applied in phylogenetic studies [12, 40]. For the coding regions, the most divergent regions were *ycf1*, *ycf2*, *rpl20*, and *rpoc2*. These are called hotspot regions as they contain clustered variation such as single-nucleotide polymorphisms and indels [48, 49]. The cp genomes of *Aconitum* species also contain general hotspot regions for variation similar to those of other plants. These divergent regions of *Aconitum* species could be developed as genomic information for development of molecular markers for use in DNA barcoding and phylogenic analysis in *Aconitum* species.

### Phylogenic relationship of *Aconitum* species within Ranunculaceae

**Fig 5:**
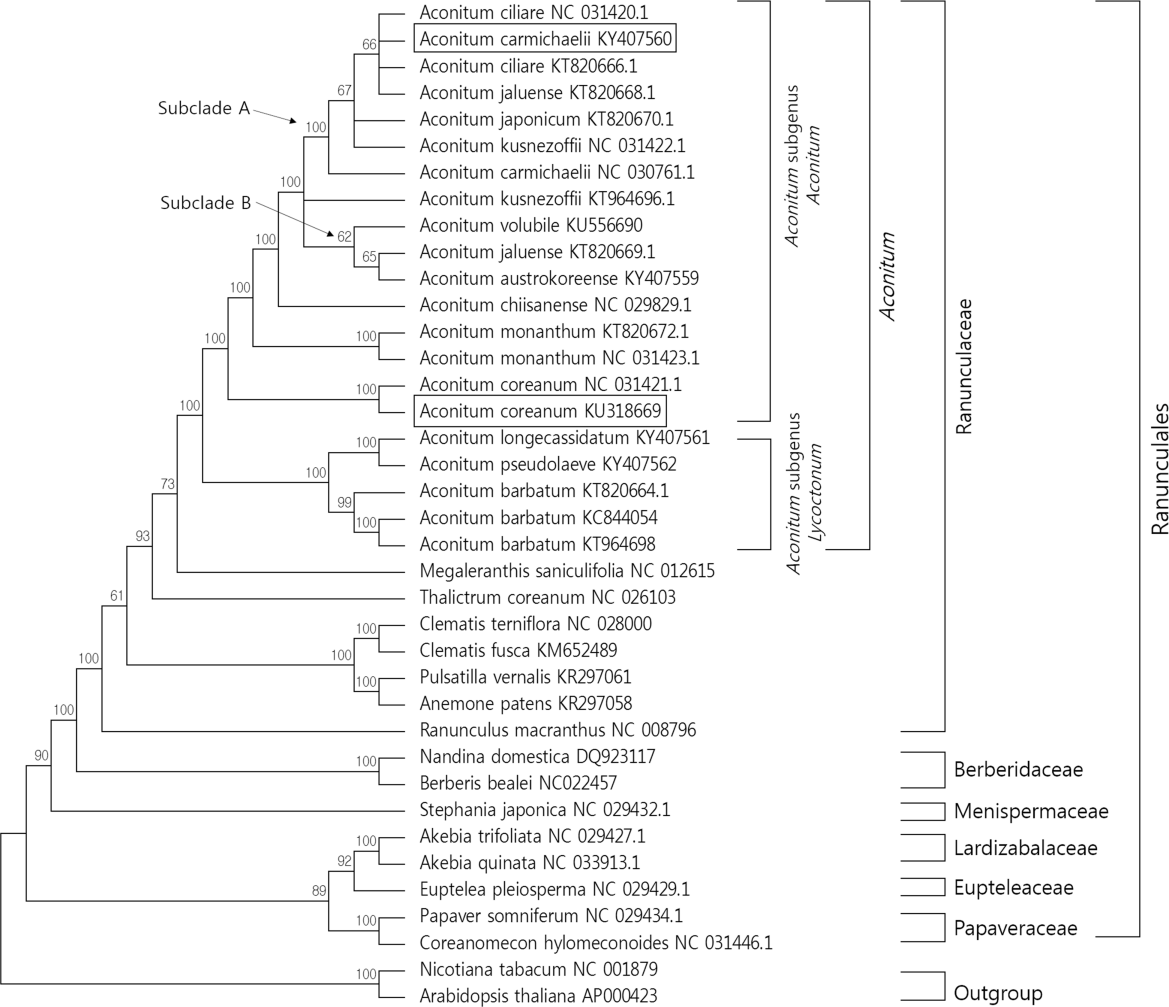
Molecular phylogenetic tree of 38 plants including 21 *Aconitum* species based on 70 protein-coding genes in the cp genome. The tree was constructed by maximum likelihood analysis using MEGA6 with a bootstrap test of 1,000 replications.

To identify the phylogenetic positon of *Aconitum* species within Ranunculaceae and their relationship to other families within Ranunculales, 70 protein-coding sequences shared by the 38 cp genomes were aligned (Fig 5). Two species, *Nicotiana tabacum* (NC001879) and *Arabidopsis thaliana* (AP000423), were set as outgroups. Maximum likelihood (ML) analysis was performed with 1,000 bootstrap replicates. The reconstructed phylogeny showed that 21 nodes had bootstrap values >95%, and 19 of these had bootstrap values of 100%. The clusters consisting of 38 species, including 21 *Aconitum* species, were well supported in six families; Ranunculaceae, Berberidaceae, Menispermaceae, Lardizabalacea, Euptrleaceae, and Papaveraceae in Ranunculales. Ranunculaceae and Berberidaceae are the closest families according to the APG III system of flowering plant classification [50] (Fig 5). *Aconitum* species were strongly clustered, and *M*. *saniculifilia* and *T*. *coreanum* were closer than other species in Ranunculaceae. Twenty-one *Aconitum* species formed a clade as two subgenera, *Aconitum* subgen. *Aconitum* and *Aconitum* subgen. *Lycoctonum*. *A*. *barbatum* clustered with *A*. *longecassidatum* and *A*. *pseudolaveve* in the subgenus *Lycoctonum* [51]. In *Aconitum* subgen. *Aconitum*, *A*. *ciliare* (NC031420), *A*. *carmichaelii* (KY407560), *A*. *ciliare* (KT820666), *A*. *jaluense* (KT820668), *A*. *japonicum* (KT820670), *A*. *kusnezoffii* (NC031422), and *A*. *carmichaelii* (NC030761) clustered monophyletically (subclade A) with a bootstrap value of 100%. Another monophyletic subclade (subclade B) also formed a cluster and included *A*. *volubile* (KU556690), *A*. *jaluense* (KT820669), *A*. *austrokoreense* (KY407559), with a weak bootstrap value of 62%. *A*. *kusnezoffii* (KT964696) clustered paraphyletically with subclade A and B. In addition, *A*. *jaluense* was included in both subclades (subclade A and B) (Fig 5). To verify the reasons for the polyphyletic division of the two species, *A*. *kusnezoffii* and *A*. *jaluense*, we critically analyzed the coding sequences by performing sequence alignments. Interestingly, we identified diverse intra-species specific sequence variabilities for *A*. *kusnezoffii*, and *A*. *jaluense*, but not for other *Aconitum* species such as *A*. *ciliare*, *A*. *monanthum*, and *A*. *coreanum*. Thus, we estimate that the two subclades separated by weak bootstrap values branched via intra-specific variation of *A*. *kusnezoffii* and *A*. *jaluense* cp genomes in each species. To identify the exact position of *A*. *kusnezoffii* and *A*. *jaluense*, we additionally confirmed phylogenic relationships based on one accession of each *A*. *kusnezoffii* and *A*. *jaluense*, *A*. *kusnezoffii* (NC031422) and *A*. *jaluense* (KT820668) (S2A Fig) and *A*. *kusnezoffii* (KT964696) and *A*. *jaluense* (KT820669) (S2B Fig), respectively.

The results showed that the positions of *A*. *kusnezoffii* and *A*. *jaluense* were unchanged in the *Aconitum* subgen. *Aconitum* (S2 Fig). It has been reported that *Aconitum* subgen. *Aconitum* exist as diploid, tetraploid, and hexaploid varieties in East Asia, of which the tetraploid variety is the most common. [52]. Interestingly, *A*. *kusnezoffii* and *A*. *jaluense*, *A*. *carmichaelii*, *A*. *japonicum*, and *A*. *ciliare* were reported to be tetraploids in a previous report [52, 53]. Polyploidization is an evolutionally common event in natural populations [54, 55]. Two different accessions of *A*. *ciliare* (NC 031420 and KT820666) clustered monophyletically (Fig 5) and seemed to have the same ploidy and maternal inheritance pattern. However, four *Aconitum* species (*A*. *kusnezoffii* and *A*. *jaluense*, *A*. *carmichaelii*, and *A*. *japonicum*) having different accessions from the same species did not clearly cluster monophyletically (Fig 5). We inferred that the *Aconitum* accessions of the four species had different ploidy even if they correspond to the same species reported previously [52]. As shown Fig 5, polyphyletic relationship of different individuals from the same species including *A*. *kusnezoffii* and *A*. *jaluense* seem to be depending on ploidy within the species.

Taken together, the results suggest that the species identification of *Aconitum* subgen. *Aconitum* is limited depending on cp genome sequence in several taxa because of the low inter-species variability and high intra-species variability.

In previous studies, several phylogenetic trees were constructed to verify the relationships between species of *Aconitum* subgen. *Aconitum* based on ITS, *trnL- F* sequences [53]. ITS sequence-based phylogenetic analysis of 52 species of *Aconitum* subgen. *Aconitum* classified them into two seed groups. In addition, *A*. *austrokoreense* and *A*. *monanthum* formed a paraphyly with *A*. *variegatum*, *A*. *napellus*, and unidentified *Aconitum* samples using ITS sequences [53]. Likewise, in cp phylogenetic analysis, *A*. *monanthum* and *A*. *austrokoreense* were consistent with previous study. *A*. *carmichaelii* (KY407560) and *A*. *coreanum* (KU318669) showed a polyphyletic relationship in *Aconitum* subgen. *Aconitum* (Fig 5). These results indicate that phylogenetic analysis of cp genome sequences could provide useful information for analysing the phylogenetic relationships between *Aconitum* subgen. *Aconitum* species. However, the results suggest that additional largescale genomic analyses using numerous and accurately identified *Aconitum* species will be required to clarify the taxonomy and phylogenetic relationships of *Aconitum* subgen. *Aconitum* at low taxonomic levels, including species identification.

### Authentication of *A*. *coreanum* using a SCAR marker

**Fig 6:**
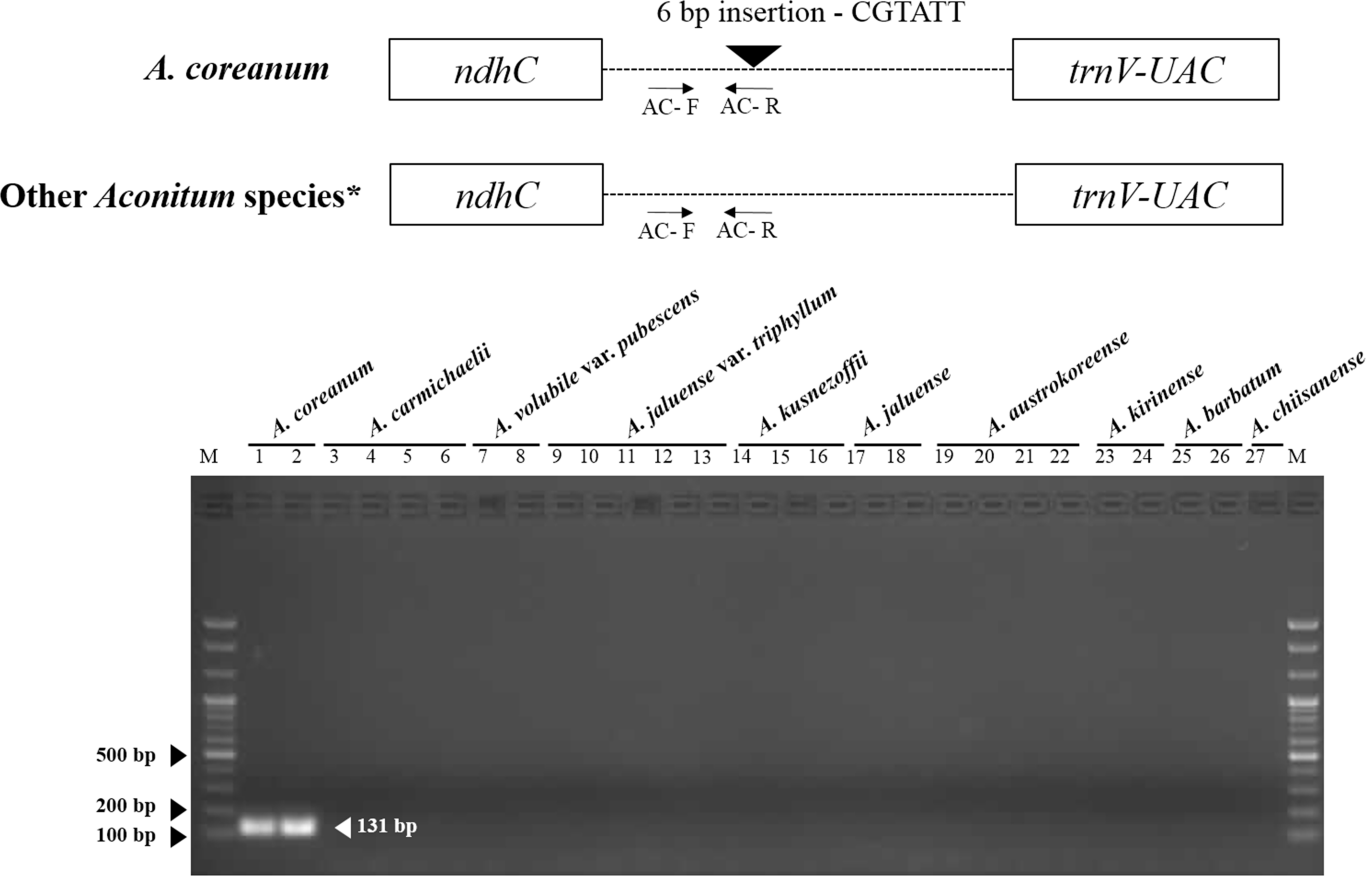
Schematic diagram of the indel region and development of *A*. *coreanum*-specific SCAR marker for the identification of Aconiti Koreani Tuber. 1–2. *A*. *coreanum*; 3–6. *A*. *carmichaelii*; 7–8 *A*. *voluvile* var. *pubescens*; 9–13. *A*. *jaluense* var. *triphyllum*; 14–16. *A*. *kusnezoffii*; 17–18. *A*. *jaluense*; 19–22. *A*. *austrokoreense*; 23–24. *A*. *kirinense*; 25–26. *A*. *barbatum*; 27. *A*. *chiisanense*; M. 100bp DNA ladder. * 6 taxa of *Aconitum* species were used in this study.

In comparative analysis of the complete cp genome sequences of *Aconitum* species, we obtained a highly divergent and specific sequence with an indel mutation only in the *ndhC*–*trnV* region of *A*. *coreanum*. Firstly, Sanger sequencing of the *ndhC*–*trnV* region and sequence alignment were performed using multiple accessions of germplasm to determine whether or not *A*. *coreanum*-specific sequence variation is detected only in *A*. *coreanum*. The results showed that *A*. *coreanum* had a 6 bp insertion that was absent in the other *Aconitum* species (S3 Fig). Based on these specific sequences, specific primers for amplifying SCAR were designed and those specifies were confirmed (Fig 6). To confirm the specificity of SCAR primers, we employed a set of nine species and 1 variety in the genus *Aconitum* consisting of a total of 27 samples (Fig 6 and Table 4). PCR amplification resulted in a DNA fragment with the expected SCAR amplicon size from *A*. *coreanum* (131 bp), whereas no PCR product was observed for other *Aconitum* species (Fig 6). Thus, we were successful in obtaining a species-specific SCAR marker for *A*. *coreanum*.

**Table 4:** Primers for SCAR marker development.

Primer name	Primer sequence (5' > 3')	Product size	Position
AC-F	GCC AAA ATA GGA ATA ACA CTT GAT ATT	131 bp	*ndhC*–*trnV* (IGS)
AC-R	GAA TCG ACT AAT ACG AAT ACG GAT		

In a previous study, 19 *Aconitum* species were classified into ten subgroups based on sequence variabilities in the *psbA-trnH* region. Of these, *A*. *camichaelii* and *A*. *kusnezoffii* had the same nucleotide sequences, whereas *A*. *coreanum* showed sequence variability with respect to those of other *Aconitum* species [16]. However, our study analyzing the same sequences of nine *Aconitum* species found that *A*. *coreanum* could not be differentiated from either *A*. *volubile* or *A*. *austrokoreense*. Thus, sequence variability in the *psbA-trnH* region could not differentiate *A*. *coreanum* from *A*. *volubile* or *A*. *austrokoreense* at the species level because they had the same sequences in our preliminarily analysis. Furthermore, to develop a genetic authentication tool for *Aconitum*-based herbal medicines, we analyzed DNA barcode regions (*ITS*, *matK*, and *psbA-trnH*) in *Aconitum* germplasms. We observed several nucleotide substitutions that could be used to identify *A*. *coreanum* and *A*. *kiriensis* from other *Aconitum* species using comparative analysis of entire sequences of the nrDNA-ITS regions. However, the number of nucleotide substitutions in the nrDNA-ITS regions was not enough to develop a SCAR marker, and *matK* gene sequences did not show any sequences differences similar to those found in the *psbA-trnH* region. Therefore, we conducted further comparative analysis of complete cp genome sequences to overcome the limitations of the universal DNA barcode used previously to distinguish *Aconitum* species. Since *Aconitum* cp genomes have high similarity (97.36–99.9%), it was difficult to develop species-specific markers for each *Aconitum* species. Although plants of *Aconitum* species show morphological differences in their areal parts, identification of the botanical origin of the dried tubers used in herbal medicines is very difficult. In this study, we developed an *A*. *coreanum-*specific SCAR marker using ten *Aconitum* taxa. However, further study will be required to distinguish between different Aconiti herbal medicines employing *Aconitum* species and to identify the botanical origins at the species level for quality control purposes. The SCAR marker developed in this study will help authenticate rapidly and accurately herbal medicines, and determine whether they have been adulterated or not.

## Conclusions

In this paper, we obtained the complete chloroplast sequences of *A*. *coreanum* and *A*. *carmichaelii* belonging to the Ranunculaceae family. The two *Aconitum* species had slightly different genome sizes, a feature shared by other *Aconitum* species. Furthermore, the distributions and locations of SSRs were determined. The cp genome sequences were compared with those of six other *Aconitum* species. The most divergent regions were found in the *trnK-UUU–trnQ-UUG*, *trnS-GCU–trnG-GCC*, *petN*–*psbM*, *ndhC–trnV-UAC*, *ycf4*–*cemA*, *rpl18*–*rpl20*, and *trnT*–*psbD* non-coding regions, and in the *ycf1*-, *ycf2*-, *rpl20*-, and *rpoc2*-coding regions. A phylogenetic analysis was performed on the 20 cp genomes of 13 *Aconitum* species with the cp genomes of five other families within the Ranunculales. We also developed a SCAR marker for *A*. *coreanum* using a divergent region of the *Aconitum* cp genome. These results provide useful information for phylogenetic studies on the Ranunculaceae and the biological systematics of *Aconitum* L., as well as for the conservation biology of *A*. *coreanum*. Furthermore, the SCAR maker could be useful in distinguishing *A*. *coreanum* from other *Aconitum* species, particularly with respect to the authentication of herbal medicines.

## Supporting information

S1 Fig Assembly of the complete chloroplast genome sequences of *Aconitum* species. (PDF)

S2 Fig Molecular phylogenetic tree of 19 *Aconitum* species based on 70 protein-coding genes in the cp genome. (PDF)

S3 Fig Comparative analysis of the *ndhC*–*trnV* region (IGS) in six *Aconitum* species. (PDF)

S1 Table GenBank accession numbers of the cp genomes used in this study. (PDF)

S2 Table Raw reads and trimmed read data. (PDF)

S3 Table *Aconitum* cp genome assembly information. (PDF)

S4 Table The genes with introns in the chloroplast genomes of *Aconitum* species and the lengths of exons and introns. (PDF)

S5 Table Codon-anticodon recognition pattern and codon usage for *Aconitum* cp genomes. (PDF)

S6 Table Base composition of the chloroplast genomes of two *Aconitum* species. (PDF)

S7 Table Distribution of tandem repeats in the cp genomes of two *Aconitum* species. (PDF)
