# Supplementary material for: The complete chloroplast genome sequence of Aconitum coreanum and Aconitum carmichaelii and comparative analysis with other Aconitum species
Source: PLoS One. 2017 Sep 1;12(9):e0184257. doi: 10.1371/journal.pone.0184257 (PMC5581188; doi:10.1371/journal.pone.0184257)
Supplement: S1 Fig — (PDF) [file pone.0184257.s001.pdf]

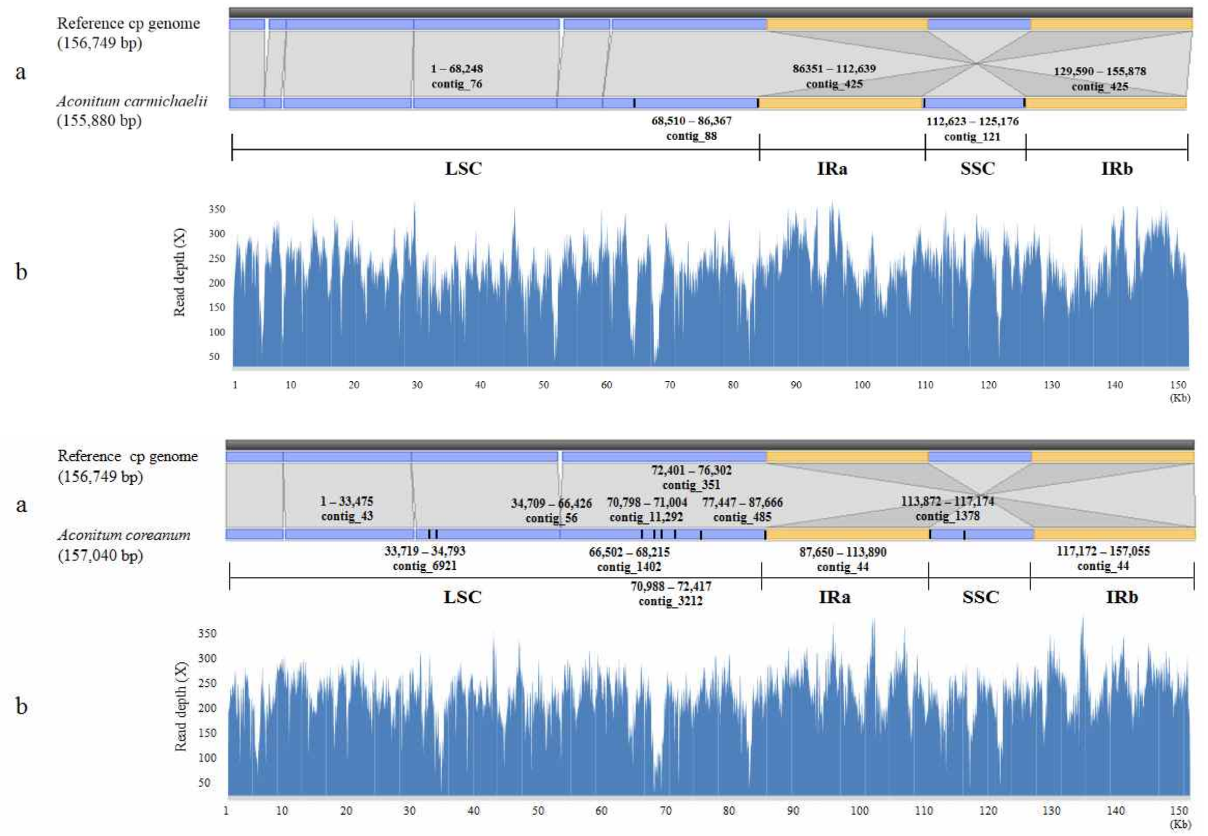

S1 Fig. Assembly result of the complete chloroplast genome sequence of *Aconitum* species. (A) Our assembly result validated to the reference genome of *A. barbatum* var. *puberulum* (GenBank accession KC844054) using BLASTZ analysis. Alignment of initial contigs is represented on the complete cp genome. The position of matching contigs is denoted at the complete genome sequence. Reference genome and assembly are compared with corresponding regions. (B) Mapping of Paired-end reads to the completed cp genome of *Aconitum* species is shown.
