# Supplementary material for: The complete chloroplast genome sequence of Aconitum coreanum and Aconitum carmichaelii and comparative analysis with other Aconitum species
Source: PLoS One. 2017 Sep 1;12(9):e0184257. doi: 10.1371/journal.pone.0184257 (PMC5581188; doi:10.1371/journal.pone.0184257)
Supplement: S2 Fig — (PDF) [file pone.0184257.s002.pdf]

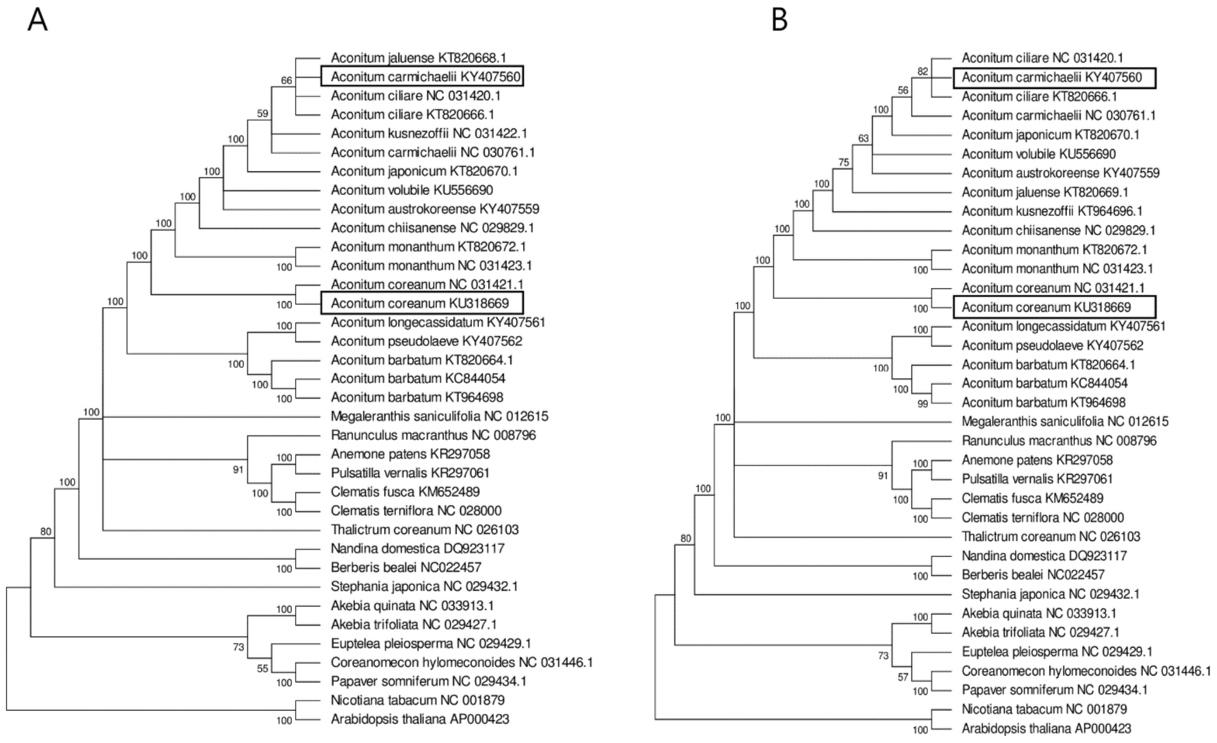

S2 Fig. Molecular phylogenetic tree of 19 *Aconitum* species based on 70 protein-coding genes in the cp genome. (A) 19 *Aconitum* species used in this study excluding *A. kusnezoffii* (KT964696.1) and *A. jaluenese* (KT 820669.1). (B) 19 *Aconitum* species used in this study excluding *A. kusnezoffii* (NC 031422.1) and *A. jaluenese* (KT820668.1). The tree was constructed by maximum likelihood analysis using MEGA6 with a bootstrap test of 1,000 replications.
