# Supplementary material for: The complete chloroplast genome sequence of Aconitum coreanum and Aconitum carmichaelii and comparative analysis with other Aconitum species
Source: PLoS One. 2017 Sep 1;12(9):e0184257. doi: 10.1371/journal.pone.0184257 (PMC5581188; doi:10.1371/journal.pone.0184257)
Supplement: S1 Table — (PDF) [file pone.0184257.s004.pdf]

S1 Table. GenBank accession numbers of the cp genomes used in this study.

Order	No.	Family	Taxon	GenBank Accession number
Ranunculales	1	Berberidaceae	*Nandiana domestica*	DQ_923117
	2	Berberidaceae	*Berberis bealei*	NC_022457
	3	Menispermaceae	*Stephania japonica*	NC_029432.1
	4	Lardizabalaceae	*Akebia trifoliata*	NC_029427.1
	5	Lardizabalaceae	*Akebia quinata*	NC_033913.1
	6	Eupteleaceae	*Euptelea pleiosperma*	NC_029429.1
	7	Papaveraceae	*Coreanomecon hylomeconoides*	NC_031446.1
	8	Papaveraceae	*Papaver somniferum*	NC_029434.1
	9	Ranunculaceae	*Megaleranthis saniculifolia*	NC_012615
	10		*Thalictrum coreanum*	NC_026103
	11		*Clematis fusca var. coreana*	KM_652489
	12		*Anemone patens*	KR_297058
	13		*puldatilla vernalis*	KR_297061
	14		*Ranunculus macranthus*	NC_008796
	15		*Clematis terniflora*	NC_028000
	16		*Aconitum barbatum var. puberulum*	KC_844054
	17		*Aconitum chiisanense*	NC_029829.1
	18		*Aconitum japonicum subsp. napiforme*	KT_820670.1
	19		*Aconitum jaluense subsp. jaluense*	KT_820669.1
	20		*Aconitum barbatum var. puberulum*	KT_964698.1
	21		*Aconitum barbatum var. hispidum*	KT_820664.1
	22		*Aconitum monanthum*	NC_031423.1
	23		*Aconitum kusnezoffii*	NC_031422.1
	24		*Aconitum kusnezoffii*	KT_964696.1
	25		*Aconitum coreanum*	NC_031421.1
	26		*Aconitum ciliare*	NC_031420.1
	27		*Aconitum monanthum*	KT_820672.1
	28		*Aconitum jaluense subsp. jaluense*	KT_820668.1
	29		*Aconitum ciliare*	KT_820666.1
	30		*Aconitum carmichaelii*	NC_030761.1
	31		*Aconitum volubile*	KU_556690
	32		*Aconitum austrokoreense*	KY_407559
	33		*Aconitum pseudolaeve*	KY_407562
	34		*Aconitum longecassidatum*	KY_407561
outgroup asterids	35	Solanaceae	*Nicotiana tabacum*	NC_001879.2
outgroup rosids	36	Brassicaceae	*Arabidopsis thaliana*	AP000423.1
