# Supplementary material for: The complete chloroplast genome sequence of Aconitum coreanum and Aconitum carmichaelii and comparative analysis with other Aconitum species
Source: PLoS One. 2017 Sep 1;12(9):e0184257. doi: 10.1371/journal.pone.0184257 (PMC5581188; doi:10.1371/journal.pone.0184257)
Supplement: S2 Table — (PDF) [file pone.0184257.s005.pdf]

S2 Table. Raw read and trimmed read data.

Species	Input reads	Trimmed reads		Total raw bases	Trimmed bases	
Aconitum carmichaelii Debeaux	34,093,340	21,589,191	63.32%	5,124,848,866	2,849,320,226	55.60%
Aconitum coreanum (H.Lév.) Rapaics.	34,061,576	21,603,072	63.42%	5,145,840,765	2,846,052,166	55.57%
