# Supplementary material for: The complete chloroplast genome sequence of Aconitum coreanum and Aconitum carmichaelii and comparative analysis with other Aconitum species
Source: PLoS One. 2017 Sep 1;12(9):e0184257. doi: 10.1371/journal.pone.0184257 (PMC5581188; doi:10.1371/journal.pone.0184257)
Supplement: S3 Table — (PDF) [file pone.0184257.s006.pdf]

S3 Table. ***Aconitum*** **cp genome assembly information.**

Species	Aligned reads to the cp genome	Cp genome coverage (×)	Cp genome length (bp)	# of contigs
A. carmichaelii	244,334	206.52	155,880	5
A. coreanum	321,623	268.92	157,040	10
