# Supplementary material for: The complete chloroplast genome sequence of Aconitum coreanum and Aconitum carmichaelii and comparative analysis with other Aconitum species
Source: PLoS One. 2017 Sep 1;12(9):e0184257. doi: 10.1371/journal.pone.0184257 (PMC5581188; doi:10.1371/journal.pone.0184257)
Supplement: S4 Table — (PDF) [file pone.0184257.s007.pdf]

S4 Table. The genes with introns in the chloroplast genomes of *Aconitum* species and the lengths of exons and introns.

A. carmichaelii	Region	Exon I	Intron I	Exon II	Intron II	Exon III
trnk-UUU	LSC	37	2523	35		
trnG-UCC	LSC	23	721	48		
atpF	LSC	145	731	410		
rpoC1	LSC	432	769	1611		
ycf3	LSC	124	722	230	765	153
trnL-UAA	LSC	35	495	50		
trnV-UAC	LSC	39	592	37		
*rps12**	LSC	114	-	232	-	26
clpP	LSC	71	847	292	676	246
petB	LSC	6	817	642		
petD	LSC	8	722	496		
rpl16	LSC	9	1123	399		
rpl2	IR	391	667	434		
ndhB	IR	777	702	756		
trnI-GAU	IR	42	937	35		
trnA-UGC	IR	38	802	35		
ndhA	SSC	553	1009	539		
A. coreanum						
trnk-UUU	LSC	37	2527	35		
trnG-UCC	LSC	23	720	48		
atpF	LSC	145	726	410		
rpoC1	LSC	432	768	1611		
ycf3	LSC	124	726	230	759	153
trnL-UAA	LSC	35	503	50		
trnV-UAC	LSC	39	602	37		
rps12	LSC	114	-	232	-	26
clpP	LSC	71	883	292	676	246
petB	LSC	6	811	642		
petD	LSC	8	705	496		
rpl16	LSC	9	1120	399		
rpl2	IR	391	667	434		
ndhB	IR	777	702	756		
trnI-GAU	IR	42	936	36		
trnA-UGC	IR	38	797	35		
ndhA	SSC	553	1014	539		

**rps12* gene is a trans-splicing gene.
