# Supplementary material for: The complete chloroplast genome sequence of Aconitum coreanum and Aconitum carmichaelii and comparative analysis with other Aconitum species
Source: PLoS One. 2017 Sep 1;12(9):e0184257. doi: 10.1371/journal.pone.0184257 (PMC5581188; doi:10.1371/journal.pone.0184257)
Supplement: S5 Table — (PDF) [file pone.0184257.s008.pdf]

S5 Table. Codon-anticodon recognition pattern and codon usage for *Aconitum* cp genomes.

Amino acid	Codon	*A. carmichaelii*		*A. coreanum*		tRNA
		Count	RSCU*	Count	RSCU	
Phe	UUU	933	1.27	937	1.27	
Phe	UUC	534	0.73	536	0.73	*trnF-GAA*
Leu	UUA	835	1.81	833	1.81	*trnL-UAA*
Leu	UUG	587	1.27	584	1.27	*trnL-CAA*
Leu	CUU	567	1.23	565	1.23	
Leu	CUC	197	0.43	199	0.43	
Leu	CUA	386	0.84	385	0.84	*trnL-UAG*
Leu	CUG	194	0.42	189	0.41	
lle	AUU	1084	1.45	1077	1.44	
lle	AUC	456	0.61	466	0.62	*trnl-GAU*
lle	AUA	703	0.94	696	0.93	*trnl-CAU*<br>*trn(f)M-CAU*<br>*trnM-CAU*
Met	AUG	637	1	641	1	*trnM-CAU*
Val	GUU	528	1.47	529	1.46	
Val	GUC	164	0.46	163	0.45	*trnV-GAC*
Val	GUA	547	1.52	551	1.52	*trnV-UAC*
Val	GUG	202	0.56	204	0.56	
Ser	UCU	568	1.68	562	1.67	
Ser	UCC	340	1	344	1.02	*trnS-GGA*
Ser	UCA	417	1.23	415	1.23	*trnS-UGA*
Ser	UCG	192	0.57	192	0.57	
Pro	CCU	427	1.5	427	1.5	
Pro	CCC	223	0.78	221	0.78	*trnS-GCU*
Pro	CCA	337	1.18	342	1.2	
Pro	CCG	152	0.53	149	0.52	
Thr	ACU	533	1.55	538	1.58	*trnP-UGG*
Thr	ACC	261	0.76	254	0.74	
Thr	ACA	420	1.22	420	1.23	
Thr	ACG	159	0.46	154	0.45	*trnT-GGU*
Ala	GCU	598	1.71	598	1.72	*trnT-UGU*
Ala	GCC	228	0.65	229	0.66	
Ala	GCA	394	1.13	395	1.13	
Ala	GCG	179	0.51	172	0.49	
Tyr	UAU	773	1.59	768	1.58	*trnA-UGC*
Tyr	UAC	202	0.41	203	0.42	
Stop	UAA	40	1.4	39	1.36	
Stop	UAG	25	0.87	25	0.87	*trnY-GUA*
His	CAU	513	1.51	513	1.5	
His	CAC	168	0.49	169	0.5	
Gln	CAA	693	1.5	691	1.51	

Gln	CAG	228	0.5	222	0.49	
Asn	AAU	989	1.53	986	1.54	*trnH-GUG*
Asn	AAC	301	0.47	297	0.46	*trnQ-UUG*
Lys	AAA	1004	1.45	997	1.45	
Lys	AAG	383	0.55	382	0.55	
Asp	GAU	886	1.6	886	1.6	*trnN-GUU*
Asp	GAC	222	0.4	222	0.4	*trnK-UUU*
Glu	GAA	1007	1.46	1013	1.47	
Glu	GAG	373	0.54	368	0.53	
Cys	UGU	227	1.46	226	1.46	*trnD-GUC*
Cys	UGC	83	0.54	84	0.54	*trnE-UUC*
Stop	UGA	21	0.73	22	0.77	
Trp	UGG	479	1	477	1	
Arg	CGU	360	1.33	362	1.35	*trnC-GCA*
Arg	CGC	94	0.35	91	0.34	*trnW-CCA*
Arg	CGA	363	1.35	360	1.34	*trnR-ACG*
Arg	CGG	117	0.43	116	0.43	
Ser	AGU	395	1.17	392	1.17	
Ser	AGC	118	0.35	113	0.34	
Arg	AGA	502	1.86	505	1.88	*trnR-UCU*
Arg	AGG	182	0.67	180	0.67	
Gly	GGU	601	1.34	606	1.34	
Gly	GGC	185	0.41	182	0.4	*trnG-GCC*
Gly	GGA	721	1.6	724	1.6	*trnG-UCC*
Gly	GGG	293	0.65	299	0.66	

*RSCU – Relative synonymous codon usage.
