# Supplementary material for: The complete chloroplast genome sequence of Aconitum coreanum and Aconitum carmichaelii and comparative analysis with other Aconitum species
Source: PLoS One. 2017 Sep 1;12(9):e0184257. doi: 10.1371/journal.pone.0184257 (PMC5581188; doi:10.1371/journal.pone.0184257)
Supplement: S6 Table — (PDF) [file pone.0184257.s009.pdf]

S6 Table. Base composition of the chloroplast genomes of two *Aconitum* species

A. carmichaelii	A (%)	T (%)	G (%)	C (%)	length (bp)
LSC	31.4	32.4	17.7	18.5	86,348
SSC	34.0	33.3	15.2	17.5	16,946
IR	28.5	28.5	21.5	21.5	52,586
Total	30.7	31.2	18.7	19.4	155,888
CDS	30.6	31.1	20.4	17.9	79,590
First position	30.6	24	26.9	18.9	26,530
Second position	29.4	32	17.9	20.5	26,530
Third position	31.6	38	16.5	14.2	26,530
A. coreanum					
LSC	31.5	32.5	17.7	18.4	87,628
SSC	34.1	33.2	15.2	17.4	16,924
IR	28.5	28.5	21.5	21.5	52,488
Total	30.8	31.2	18.7	19.3	157,040
CDS	30.5	31.2	20.4	17.9	79,461
First position	30.6	24	27.0	18.9	26,487
Second position	29.4	32	17.9	20.4	26,487
Third position	31.7	38	14.2	16.4	26,487
