# Supplementary material for: The complete chloroplast genome sequence of Aconitum coreanum and Aconitum carmichaelii and comparative analysis with other Aconitum species
Source: PLoS One. 2017 Sep 1;12(9):e0184257. doi: 10.1371/journal.pone.0184257 (PMC5581188; doi:10.1371/journal.pone.0184257)
Supplement: S3 Fig — (PDF) [file pone.0184257.s003.pdf]

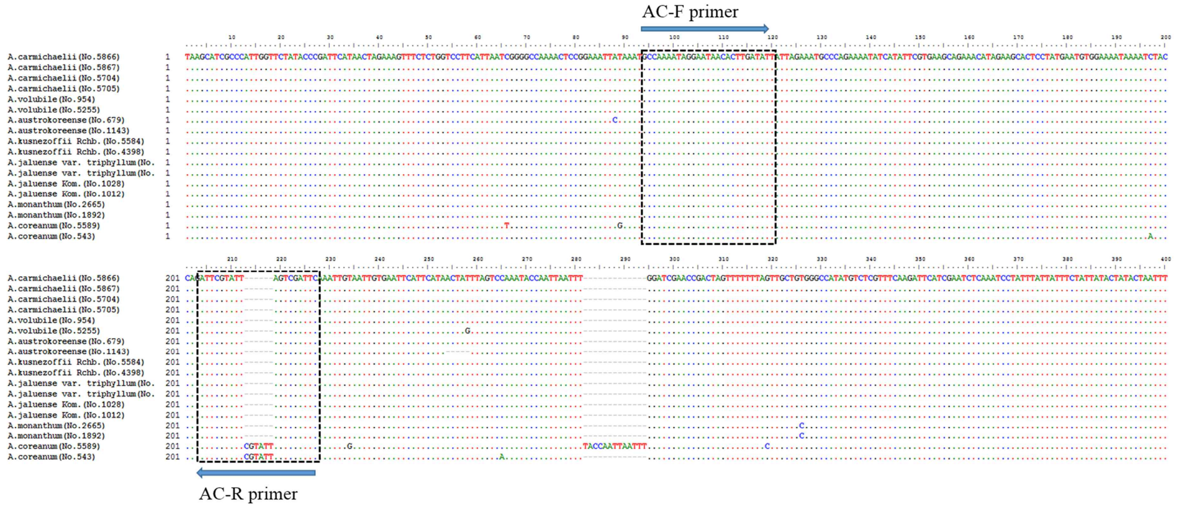

Figure S3. Comparative analysis of *ndhC–trnV* region (IGS) using 6 *Aconitum* species. The *A. coreanum*-specific primer were designed using 6 bp insertion. Parentheses indicate KIOM voucher number.
