# Supplementary material for: The complete chloroplast genome sequence of Aconitum coreanum and Aconitum carmichaelii and comparative analysis with other Aconitum species
Source: PLoS One. 2017 Sep 1;12(9):e0184257. doi: 10.1371/journal.pone.0184257 (PMC5581188; doi:10.1371/journal.pone.0184257)
Supplement: S7 Table — (PDF) [file pone.0184257.s010.pdf]

S7 Table. Distribution of tandem repeats in the cp genomes of two *Aconitum* species.

Tandem repeat *(A. carmichaelii)*	Position	Repeat unit length (bp)	Repeat unit sequence	Repeat numbers	Region
1	IGS (*trnK-UUU, rps16*)	13	TTTTTCAAGTAAA	2	LSC
2	IGS (*trnK-UUU, rps16*)	19	TATAGTTATAGTATATATA	2	LSC
3	IGS (*trnS-GCU, trnG-UCC*)	19	TACACATGAAGTAAAGAAA	2	LSC
4	IGS (*atpF, atpH*)	20	TATTGTAGGAGTGAAATCGT	2	LSC
5	Intron (*rpoC1*)	20	ACGAGGTACAACTAGTAATT	2	LSC
6	IGS (*rpoB, trnC-GCA*)	21	TGGATCGATCTAGTAATGATG	2	LSC
7	IGS (*trnT-UGU, trnL-UAA*)	23	TATGTAACATAAAGAAAATATAA	2	LSC
8	Exon (*petG*)	17	TTAAGTAACATCTCTTT	2	LSC
9	IGS (*rps18, rpl20*)	18	TGTATTCCCTCCCTTATC	2	LSC
10	Intron (*clpP*)	13	TTTTATAGTTAAA	2	LSC
11	Intron (*petB*)	18	ATTTCATCTATTAATTGA	2	LSC
12	Exon (*rps11*)	24	ACGTCCATTCCTACGCGAGCCAAT	2	LSC
13	Exon (*ycf2*)	15	AAGAGGATGAGCTTC	3	IR
14	Exon (*ycf1*)	15	TTAATATAATAAAAT	2	SSC
15	IGS (*rps15, ycf1*)	16	TACAATACACAAAAAT	2	SSC
16	Exon (*ycf1*)	12	TACTCCTAAAAA	2	SSC
17	Exon (*ycf2*)	15	TCATCCTCTTGAAGC	3	IR
18	Exon (*ycf2*)	18	ATCATCAAT	3	IR
**Tandem repeat (*A. coreanum*)**					
1	IGS (*trnK-UUU, rps16*)	13	TTTTTCAAGTAAA	2	LSC
2	IGS (*trnK-UUU, rps16*)	19	TATAGTTATAGTATATATA	2	LSC
3	IGS (trnS-GCU, trnG-UCC)	19	TACACATGAAGTAAAGAAA	2	LSC
4	IGS (*atpF, atpH*)	20	TATTGTAGGAGTGAAATCGT	2	LSC
5	Intron (*rpoC1*)	20	ACGAGGTACAACTAGTAATT	2	LSC
6	IGS (*rpoB, trnC-GCA*)	21	TGGATCGATCTAGTAATGATG	2	LSC
7	IGS (*trnT-UGU, trnL-UAA*)	23	TATGTAACATAAAGAAAATATAA	2	LSC
8	Exon (*petG*)	17	TTAAGTAACATCTCTTT	2	LSC
9	IGS (*rps18, rpl20*)	18	TGTATTCCCTCCCTTATC	2	LSC
10	Intron (*clpP*)	13	TTTTATAGTTAAA	2	LSC
11	Intron (*petB*)	18	ATTTCATCTATTAATTGA	2	LSC
12	Exon (*rps11*)	24	ACGTCCATTCCTACGCGAGCCAAT	2	LSC
13	Exon (*ycf2*)	15	AAGAGGATGAGCTTC	3	IR
14	Exon (*ycf1*)	15	TTAATATAATAAAAT	2	SSC
15	IGS (*rps15, ycf1*)	16	TACAATACACAAAAAT	2	SSC
16	Exon (*ycf1*)	12	TACTCCTAAAAA	2	SSC
17	Exon (*ycf2*)	15	TCATCCTCTTGAAGC	3	IR
18	Exon (*ycf2*)	18	ATCATCAAT	3	IR
